# The intergenic region of the maize defensin-like protein genes *Def1 and Def2* functions as an embryo-specific asymmetric bidirectional promoter

**DOI:** 10.1093/jxb/erw226

**Published:** 2016-06-08

**Authors:** Xiaoqing Liu, Wenzhu Yang, Ye Li, Suzhen Li, Xiaojin Zhou, Qianqian Zhao, Yunliu Fan, Min Lin, Rumei Chen

**Affiliations:** ^1^Department of Crop Genomics & Genetic Improvement, Biotechnology Research Institute, Chinese Academy of Agricultural Sciences, 12 ZhongGuanCun South Street, Beijing 100081, China; ^2^Department of Agronomy, Agricultural University of Hebei, 289 LingYuSi Avenue, Baoding 071001, China

**Keywords:** Bidirectional promoter, *cis*-elements, embryo specificity, immature embryo transient expression system, transgenic maize, truncation analysis.

## Abstract

Bidirectional promoters are identified in diverse organisms with widely varied genome sizes, including bacteria, yeast, mammals, and plants. However, little research has been done on any individual endogenous bidirectional promoter from plants. Here, we describe a promoter positioned in the intergenic region of two defensin-like protein genes, *Def1* and *Def2* in maize (*Zea mays*). We examined the expression profiles of *Def1* and *Def2* in 14 maize tissues by qRT-PCR, and the results showed that this gene pair was expressed abundantly and specifically in seeds. When fused to either green fluorescent protein (GFP) or β-glucuronidase (GUS) reporter genes, *P*_*ZmBD1*_, *P*_*ZmDef1*_, and *P*_*ZmDef2*_ were active and reproduced the expression patterns of both *Def1* and *Def2* genes in transformed immature maize embryos, as well as in developing seeds of transgenic maize. Comparative analysis revealed that P_ZmBD1_ shared most of the expression characteristics of the two polar promoters, but displayed more stringent embryo specificity, delayed expression initiation, and asymmetric promoter activity. Moreover, a truncated promoter study revealed that the core promoters only exhibit basic bidirectional activity, while interacting with necessary *cis*-elements, which leads to polarity and different strengths. The sophisticated interaction or counteraction between the core promoter and *cis*-elements may potentially regulate bidirectional promoters.

## Highlight

Here we cloned and intensively characterized the first natural embryo-specific bidirectional promoter which could facilitate multi-gene expression in maize, and proposed a model of regulation of bidirectional transcription initiation.

## Introduction

Promoters in the intergenic regions of divergent genes are arranged in one of three ways: head-to-head, tail-to-tail, or overlapping (Beck and Warren, 1988). Studies of overlapping promoters, defined as bidirectional promoters, in various species have increased in number over the past few decades. Adachi and Lieber (2002) presented an overview of bidirectional promoters in the human genome. A study reported that 10% of genes in the human genome with high GC content and less than 1kb in length shared bidirectional promoters (Trinklein *et al.*, 2004). Bidirectional promoters are widespread in yeast genomes and generate pervasive transcription (Neil *et al.*, 2009; Xu *et al.*, 2009). Krom and Ramakrishna (2008) performed a comparative analysis of the expression patterns of gene pairs whose expression was directed by a shared bidirectional promoter in rice, Arabidopsis, and *Populu*s and showed that they were mostly co-expressed and were usually involved in the same pathway. Using a bidirectional promoter search model created by Trinklein *et al.* (2004), ~2471 bidirectional promoters were identified in Arabidopsis (Wang *et al.*, 2009). Given differences in the gene organization of various species, diverse threshold values for bidirectional promoter length have been used in other plant genome-wide analyses. A more stringent criterion (<0.25kb) was used to identify 212, 462, and 141 bidirectional promoters from rice, Arabidopsis, and *Populus* (Dhadi *et al.*, 2009). Kourmpetli *et al.* (2013) coupled two criteria – a bidirectional promoter length between 0.1 and 0.6kb and the presence of at least two G-box sequences (CACGTG) within the intergenic region – to identify 70 bidirectional promoters involved in seed development in Arabidopsis. Our previous work showed that more bidirectional promoters can be identified in rice, Arabidopsis, *Populus*, soybean, and maize by using the transcript physical location instead of the gene physical location as coordinates to create a more effective search model (Liu *et al.*, 2014*b*).

Studies of bidirectional promoters on a genome-wide scale in plants are not so extensive as in animals, and fewer studies focus on individual bidirectional promoters in plants. Keddie *et al.* (1994) indicated the presence of the possible bidirectional promoter only based on gene expression in *Brassica napus*. Xie *et al.* (2001) created the first artificial plant bidirectional promoter and showed that any polar promoter could be bidirectionalized by fusing a minimal promoter in a head-to-head orientation. Subsequently, based on the same strategy artificial constitutive (Zhang *et al.*, 2008) and vascular-specific (Lv *et al.*, 2009) bidirectional promoters were generated in tobacco. A 955-bp intergenic region between the *CaTin1* and *CaTin2* genes, which directed the expression of the two genes in response to tobacco mosaic virus (TMV), was the first natural bidirectional promoter to be reported (Shin *et al.*, 2003). Two rice chymotrypsin protease inhibitor genes, *OCPI1* and *OCPI2*, share a 1126-bp natural bidirectional promoter that drove the simultaneous expression of reporter genes encoding β-glucuronidase (GUS) and green fluorescent protein (GFP) in transiently transformed onion epidermal cells (Singh *et al.*, 2009). Most studies of natural bidirectional promoters have focused on the model plant Arabidopsis. Examples of bidirectional promoters identified in Arabidopsis include two gene pairs, *cab1/cab2* and *At5g06290/At5g06280*, involved in photosynthesis which have 2177- and 351-bp bidirectional promoters, respectively (Bondino and Valle, 2009; Mitra *et al.*, 2009); 668- and 461-bp intergenic regions in the *At3g17140/At3g17150* and *At4g10596/At4g110600* gene pairs identified by characterization of two T-DNA promoter trap lines (Pratibha *et al.*, 2013; Sharma *et al.*, 2014); an asymmetric bidirectional promoter in the *At1g71850*/*At1g71860* gene pair (Liu *et al.*, 2015); and a heat-inducible bidirectional promoter in the *AtClpB-C*/*AtCK2* gene pair (Mishra and Grover, 2014).

Here, we show that two defensin-like genes are driven by a 635-bp bidirectional promoter. *Def1* and *Def2* encoded two peptides consisting of 79 and 82/121 amino acids, respectively. Fusing the *GUS* and *GFP* genes to the ends of P_ZmBD1_ and expressing the construct in transient and stable transformation systems showed that it could drive expression of the two reporter genes specifically and strongly in transgenic maize embryos. Truncation analysis of the P_ZmBD1_ region revealed that the interaction between core promoter and *cis*-elements responsible for bidirectional transcription strength and polarity.

## Materials and methods

### RNA preparation and transcript analysis

Experimental tissues, including 10 DAP (10 days after pollination) whole kernels, 15, 20, 25, and 30 DAP embryos and endosperms; and ligules, sheaths, leaves, stems, and roots of large trumpet-stage inbred line B73 maize plants were ground into powder in liquid nitrogen using a mortar and pestle. TRIzol reagent (TransGen, Beijing, China) was used to extract total RNA from the powdered tissues. Genomic DNA removal and first strand cDNA synthesis were carried out simultaneously in the same reaction system using cDNA Synthesis SuperMix (TransGen, Beijing, China). The cDNA quality was tested using maize actin gene primers. The cDNA was then used as a template for quantitative reverse transcriptase-polymerase chain reaction (qRT-PCR) using SYBR Green reagent according to the manufacturer’s instructions (TaKaRa, Dalian, China). Primers used in this work are listed in Supplementary Table S1 at *JXB* online.

### Polar and bidirectional promoter cloning and expression vector construction

Genomic sequences extending ~2.0kb upstream from the translation start sites (ATG) of the *Def1* and *Def2* genes were cloned using the high-fidelity KOD polymerase (TOYOBO, Japan). The isolated sequences were cloned into the pEASY-Blunt vector (TransGen, Beijing, China) and confirmed by sequencing. The primers used are listed in Supplementary Table S1. The 635-bp intergenic region located between the two ATG codons containing a putative bidirectional promoter was cloned and the sequence was confirmed as above. The two 2.0-kb genomic sequences and the 635-bp intergenic region were substituted for the 35S promoter located upstream of the β-glucuronidase gene (*GUS*) in the pCAMBIA3301 vector to form new expression vectors. An EGFP-polyA fragment from the pRTL2GFP vector was inserted into pCAMBIA3301 upstream of the 35S promoter in the opposite orientation to that of the *GUS* gene and the 635-bp putative bidirectional promoter and all of the truncation fragments replaced the 35S promoter to create a dual reporter gene expression vector. Promoter sequences and cloning primers are listed in Supplementary Table S1.

### Immature embryo transient expression system

Hi-II maize seeds were collected at 20 DAP for isolation of immature embryos. The peeled embryos were transferred to liquid Murashige and Skoog (MS) medium in an ultraclean hood and washed twice. The embryos were placed on solid hypertonic medium (six or nine embryos per dish) for 4h and then bombarded with particles coated with DNA constructs using a particle delivery system (Bio-Rad, Hercules, CA). The bombarded embryos were transferred to solid MS medium and incubated at 28°C for 24h in the dark, followed by detection of reporter gene expression.

### Generation of transgenic maize plants

The five expression constructs described above were used to generate transgenic maize plants by *Agrobacterium tumefaciens*-mediated transformation of immature Hi-II maize embryos (Frame *et al.*, 2002). Immature embryos 1.0–2.0mm in diameter were collected from 10–12 DAP ears, treated with 5% sodium hypochlorite solution for 30min, washed twice with sterile water, and transferred to infection medium. *A*. *tumefaciens* strain EHA105 harboring the expression vector was grown for 3 d on solid YEB medium containing rifampicin and kanamycin, scraped from the medium, transferred into the liquid infection medium containing 1% acetosyringone (AS), and cultured at 28°C with agitation at 160rpm until an OD_550_ of 0.3–0.4 was reached. Embryos were then infected with the prepared *A*. *tumefaciens* for 5min and transferred to callus induction medium followed by selection of calli resistant to 3mg l^−1^ bialaphos. Transgenic T_0_ plants were identified by PCR analysis and herbicide leaf painting.

### Detection of reporter gene activity

Histochemical staining was used for qualitative analysis of GUS activity in transiently transformed embryos and in tissues of promoter-*GUS* transgenic maize plants as described previously (Jefferson *et al.*, 1987). Tissues from bidirectional promoter-*GUS*/*GFP* transgenic maize plants were analyzed for GFP signals using a Leica fluorescence stereoscope equipped with a GFP filter followed by histochemical staining for GUS activity detection as described above. Duplicate samples of all the tissues analyzed above were collected and ground into powder in liquid nitrogen using a mortar and pestle. Total protein was isolated from the powdered tissues in extraction buffer (50mM sodium phosphate buffer, 10mM Na_2_EDTA, 1% sodium N-lauroylsarcosine, 1% Triton X-100, 0.1% beta mercaptoethanol). The GUS substrate 4-methylumbelliferyl β-D-glucuronide (4-MUG) was used for quantitative analysis. Each biological sample had three replicates.

## Results

### Structure and expression characteristics of the *Def1* and *Def2* genes in maize

**Fig. 1.**
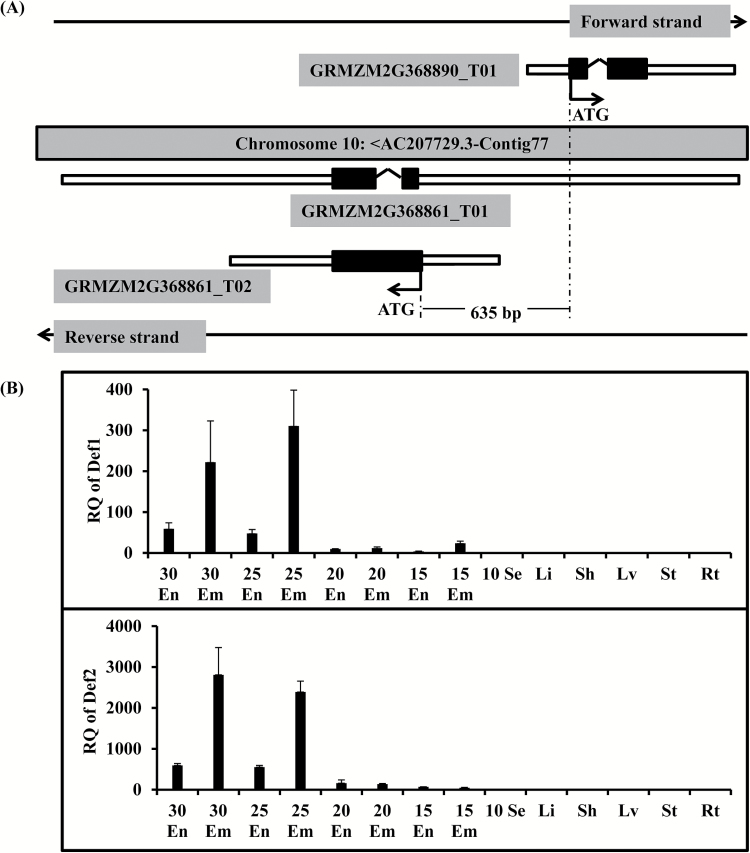
Physical location and expression characteristics of the two defensin-like protein genes *Def1* and *Def2* in maize. (A) Schematic representation of the genomic organization of the *Def1* and *Def2* genes on chromosome 10 in maize. The *Def1* gene produces only one transcript (GRMZM2G368890_T01) while the *Def2* gene produces two transcripts (GRMZM2G368861_T01 and GRMZM2G368861_T02). (B) qPCR data showing the relative transcript levels of both genes during different developmental stages of endosperm and embryos and in various vegetative tissues of large trumpet-stage inbred line B73 maize plants. En, Em, Se, Li, Sh, Lv, St, and Rt represent endosperm, embryo, seed, ligule, sheath, leaf, stem, and root tissues, respectively, of large trumpet-stage inbred line B73 maize plants; ATG, translation start site; RQ, relative expression quantity (2^–ΔΔCt^), with all the relative expression levels relative to that in root.

The genomic structure of the bidirectional gene pair is shown in Fig. 1A. The gene pair is located in contig 77 on chromosome 10. The *Def1* gene (GRMZM2G368890) transcribes one transcript from the forward strand, while the *Def2* gene (GRMZM2G368861) produces two transcripts from the reverse strand. This gene pair would be excluded by Trinklen’s model because the 5ʹ-UTR of the GRMZM2G368861_T01 transcript sequence completely overlaps the physical location of the GRMZM2G368890_T01 transcript sequence. However, under our modified model (Liu *et al.*, 2014*b*), it could be included based on the presence of the 635-bp intergenic region between the translation start sites of the GRMZM2G368861_T02 and GRMZM2G368890_T01 transcripts (Fig. 1A). According to gene annotation data of the National Center for Biotechnology Information (NCBI) database, the GRMZM2G368890 gene encodes a 79-amino acid protein named defensin-like protein 1/low-molecular-weight cysteine-rich protein (LCR68) belonging to the Knot1 super family with a putative receptor-binding site. The GRMZM2G368861 gene encodes an 82/121-amino acid protein named flower-specific gamma-thionin precursor/hypothetical protein that also belongs to the Knot1 super family. According to the gene annotation data of the Gramene and UniProt databases, GRMZM2G368890 and GRMZM2G368861 encode defensin-like protein 1/gamma-zeathionin-1 and defensin-like protein 2/gamma-zeathionin-2, respectively. We chose the names defensin-like protein 1 (DEF1) and defensin-like protein 2 (DEF2) as the protein names corresponding to the GRMZM2G368890 and GRMZM2G368861 genes for the convenience of understanding of their functions.

To confirm the expression characteristics of *Def1* and *Def2* in maize described in our previous study (Liu *et al.*, 2014*a*), we examined their expression profiles in 14 maize tissues using qRT-PCR analysis (Fig. 1B). The *Def1* and *Def2* expression profiles determined by qRT-PCR coincided with the results of our previous study (Liu *et al.*, 2014*a*). These results indicated that *Def1* and *Def2* were expressed specifically in the maize embryo and aluerone layer, and that the *Def2* expression level was higher than that of *Def1*. Moreover, the *Def1* and *Def2* expression levels were considerably higher in embryos than in endosperm.

### Functional testing of polar and bidirectional promoter activity using a maize immature embryo transient expression system

**Fig. 2.**
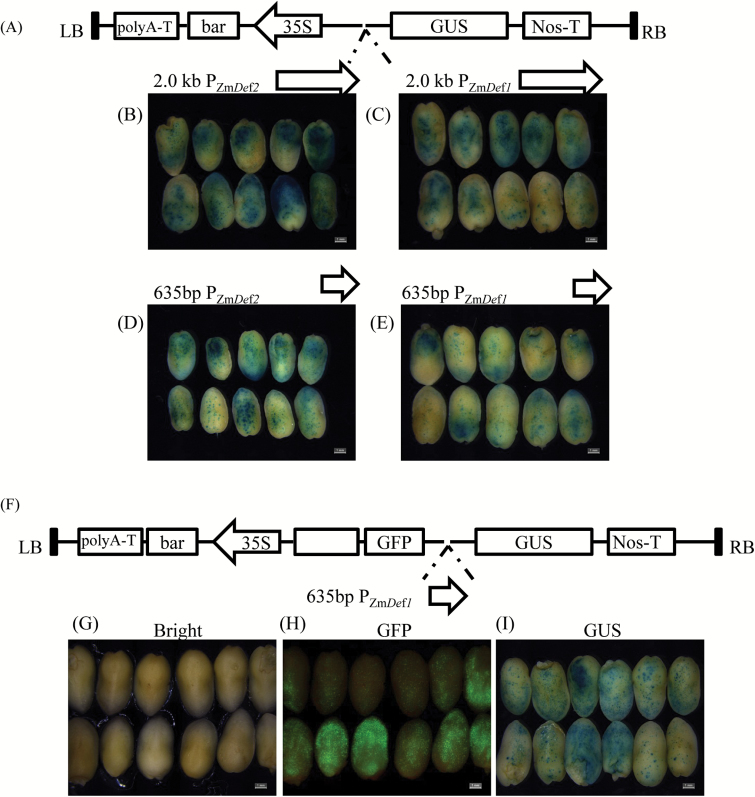
Functional analysis of various putative promoters using a maize immature embryo transient expression system. (A) Four GUS constructs that were placed under the regulation of the putative polar or bidirectional promoter of *Def1* and *Def2*, respectively. GUS staining of immature embryos bombarded with (B) the putative promoter of *Def2*, 2-kb P_*zmDef2*_::*GUS*; (C) the putative promoter of *Def1*, 2-kb P_*zmDef1*_::*GUS*; (D) 635-bp P_*zmDef2*_::*GUS*; and (E) 635-bp P_*zmDef1*_::*GUS*. (F) Diagrammatic representation of the construct containing the dual reporter genes *GFP/GUS*, driven by a 635-bp putative bidirectional promoter. Bombarded immature embryos imaged in (G) bright field mode; (H) dark field model using a GFP filter set; and in (I) bright field mode after histochemical staining. LB, left border; RB, right border; T, terminator. Bars, 0.5mm.

To verify the activities of the *Def1* and *Def2* polar promoters and to determine whether the intergenic region between the two genes functions as a bidirectional promoter, upstream sequences extending 2.0kb 5ʹ of the *Def1* and *Def2* translation start sites and the 635-bp intergenic region between the two translation start sites were amplified using PCR and inserted upstream of the *GUS* reporter gene in the pCAMBIA3301 vector to create the 635-bp P_Zm*Def1*_::*GUS*, 635-bp P_Zm*Def2*_::*GUS*, 2.0-kp P_Zm*Def1*_::*GUS*, and 2.0-kb P_Zm*Def2*_::*GUS* constructs (Fig. 2A). In addition, the 635-bp P_Zm*Def2*_ was inserted between the *GUS* and *GFP* genes contained in a dual reporter gene *GUS*/*GFP* vector derived from the pCAMBIA3301 and pRTL2GFP plasmids (Zhou *et al.*, 2013) (Fig. 2F). Twenty days after pollination, immature embryos of Hi-II maize plants were bombarded with the DNA constructs to test promoter activity using GUS staining and GFP fluorescence visualization. As shown in Fig. 2B–E, the promoters of 2.0-kb P_Zm*Def2*_ (Fig. 2B), 2.0-kb P_Zm*Def1*_ (Fig. 2C), 635-bp P_Zm*Def2*_ (Fig. 2D), and 635-bp P_Zm*Def1*_ (Fig. 2E) drove *GUS* gene expression in the bombarded maize embryos. As shown in Fig. 2G–I, the 635-bp P_Zm*Def2*_ drove simultaneous expression of the *GUS* and *GFP* reporter genes in transiently transformed maize embryos. These results indicated that the cloned 5ʹ flanking regions functioned as promoters and that the 635-bp intergenic region functioned as a bidirectional promoter.

### Comparative analysis of tissue specificity and expression levels of the polar and bidirectional promoters in transgenic maize plants

**Fig. 3.**
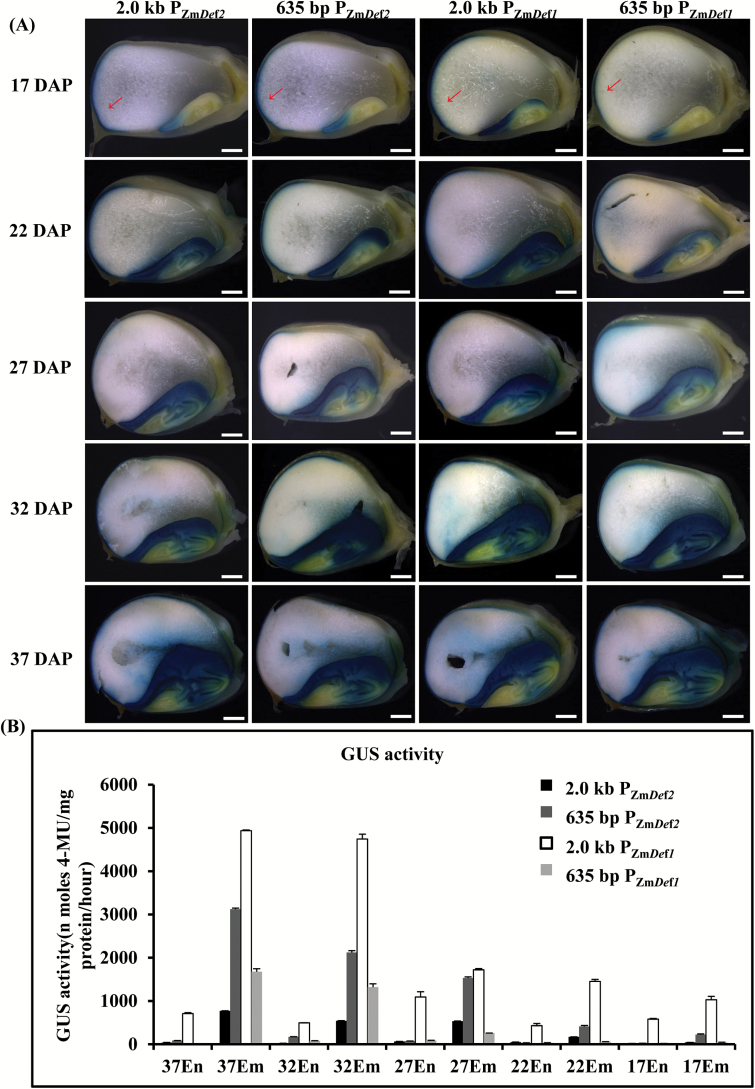
Comparative analysis of the tissue specificity and expression strength of the polar and bidirectional promoters in transgenic maize plants. (A) Analysis of promoter tissue specificity by GUS histochemical staining in transgenic maize plants. (B) GUS activities during various developmental stages of seeds from transgenic maize plants. DAP, days after pollination; En, endosperm; Em, embryo. Red arrow indicates GUS staining sites in the aleurone layer. Bars, 1mm.

To compare the tissue specificity and promoter strengths of the promoters, *GUS* or *GFP* was placed under the regulation of the 635-bp intergenic region and the 2.0-kb P_Zm*Def1*_ and P_Zm*Def2*_ polar promoters. GUS staining in diverse tissues at various developmental stages provided qualitative analysis of promoter tissue specificity and GUS activity was used to quantify promoter strength. As shown in Fig. 3A, all four promoters displayed strong expression activity in embryos at different kernel developmental stages, consistent with the results of transient expression assay shown in Fig. 2B–E. In addition, the aleurone layer showed GUS staining between 17 and 37 DAP. Moreover, the promoter strengths of the four promoters increased with seed development. The 635-bp and 2.0-kb P_Zm*Def2*_ promoters did not produce detectable GUS activity in vegetative tissues at the trefoil-stage (Supplementary Fig. S1) or in large trumpet-stage tassels, silk, or pollen of the transgenic maize plants (Supplementary Fig. S2). Like the P_Zm*Def1*_ promoters, the 635-bp P_Zm*Def1*_ also exhibited stringent embryo specificity share aluerone-specific characters (Supplementary Figs S1, S2). The 2.0-kb P_Zm*Def1*_ drove GUS expression that was induced in leaves by mechanical injury with GUS activity detected only in the knife-cut edges of the leaves, as indicated by red arrows in Supplementary Figs S1 and S2. Quantitative analysis of the GUS activities of the four promoters showed that the GUS expression levels increased during seed development (Fig. 3B). Interestingly, the strength of the 635-bp P_Zm*Def2*_ was considerably stronger than that of the 2.0-kb P_Zm*Def2*_ during all stages of embryo development, howerver the relative strengths of the 635-bp P_Zm*Def1*_ was weaker than 2.0-kb P_Zm*Def1*_ promoters. The 635-bp P_Zm*Def2*_ was stronger than the 635-bp P_Zm*Def1*_, but the 2.0-kb P_Zm*Def2*_ was markedly weaker than the 2.0-kb P_Zm*Def1*_.

To further analyze the differences between the 2.0-kb and 635-bp P_Zm*Def1*_ and P_Zm*Def2*_ promoters, we used GUS staining to analyze their expression characteristics during the early stages of seed development. As shown in Supplementary Fig. S3, initial expression of the 635-bp and 2.0-kb P_Zm*Def2*_ and the 635-bp P_Zm*Def1*_ started in the endosperm aleurone layer, while the initial expression of the 2.0-kb P_Zm*Def1*_ started in the embryo. Expression of both the 635-bp and 2.0-kb P_Zm*Def2*_ promoters in embryos began at 16 DAP with different expression levels, whereas weak expression of the 2.0-kb P_Zm*Def1*_ began at 12 DAP and expression of the 635-bp P_Zm*Def1*_ began at 17 DAP.

**Fig. 4.**
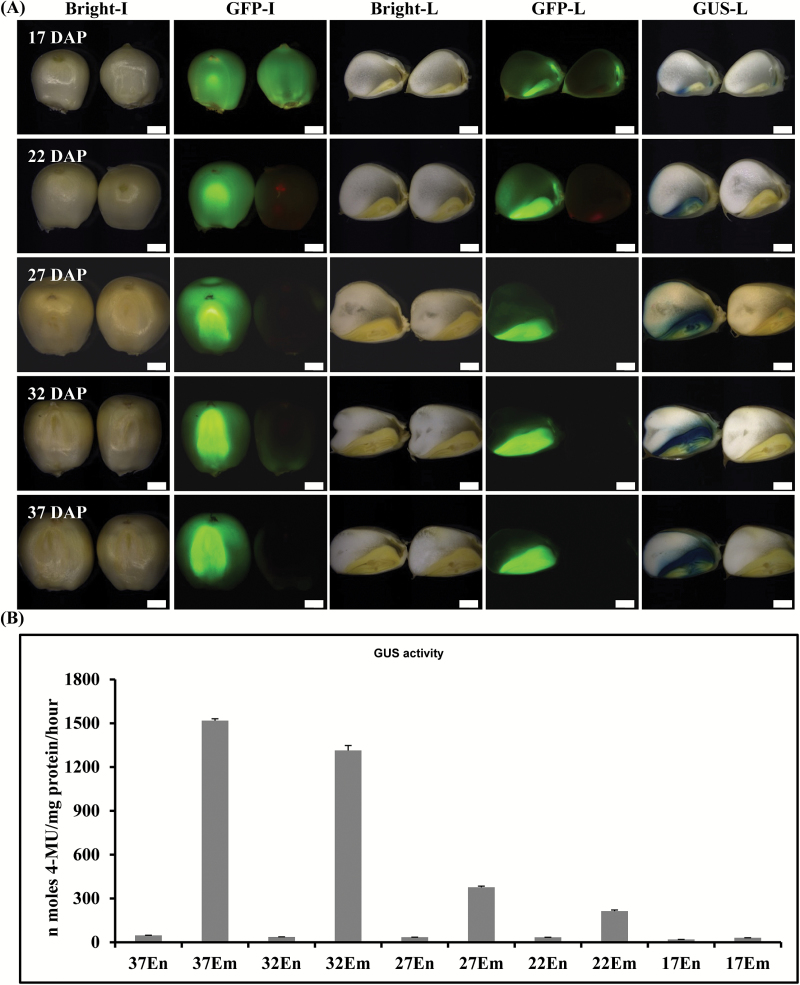
Characterization of the tissue specificity and expression strength of the P_Zmdef1_ in transgenic maize plants. (A) Qualitative analysis of bidirectional expression activities of P_Zmdef1_ in transgenic maize seeds. Bright-I and GFP-I, intact seeds imaged in bright field mode and using a GFP filter set, respectively; Bright-L, GFP-L, and GUS-L, longitudinal sections imaged in bright field mode, using a GFP filter set, and in bright field mode after histochemical staining, respectively. Control tissues corresponding to the various developmental stages are shown on the right side of each photograph. Bars, 1mm. (B) Quantification of GUS activities level during various developmental stages of seeds from transgenic maize plants. En, endosperm; Em, embryo.

The expression characteristics of the 635-bp P_Zm*Def1*_ and P_Zm*Def2*_ promoters were confirmed by analyzing the expression of the dual reporter genes in the *GFP*::P_Zm *Def1*_:: *GUS* construct (Fig. 2F) in transgenic maize plants (Fig. 4). GUS staining and quantitative analysis of GUS activity in kernels were performed as described above accompanied by GFP fluorescence visualization. As shown in Fig. 4A, P_Zmdef1_ drove *GUS* and *GFP* reporter gene expression in embryos and in the endosperm aleurone layer from 17 to 37 DAP and quantitative analysis of GUS activity in seeds showed that the expression level increased with seed development (Fig. 4B). GUS activity was not detected in non-seed tissues (Supplementary Fig. S4). The results generated by the 635-bp P_zmDef2_ with dual reporter genes in transgenic maize plant (Supplementary Fig. S5) also reproduced the expression pattern of Def1 and Def2 as P_zmDef1_ did. These results were consistent with the results obtained from transgenic maize plants expressing constructs in which the *GUS* reporter gene was fused to either end of the bidirectional promoter (Fig. 3), and the intergenic region (635-bp P_Zmdef1_ or 635-bp P_Zmdef2_) between the Zm_*Def1*_ and Zm_*Def2*_ genes functions as an embryo-specific and asymmetric bidirectional promoter: P_ZmBD1_.

Taken together, these results indicated that the 2.0-kb P_Zm*Def2*_ and P_Zm*Def1*_ were stringent embryo-specific promoters that share alerone-specific characters and the 635-bp intergenic region functions as an embryo-specific and asymmetric bidirectional promoter (P_ZmBD1_). Moreover, P_ZmBD1_ had different expression characteristics compared to the 2.0-kb polar promoters of the two genes. We designated this 635-bp bidirectional promoter P_ZmBD1_ to reflect that it is the first natural bidirectional promoter to be cloned from maize and characterized intensively.

### Truncation analysis of the *cis*-elements involved in regulating bidirectional transcription

**Fig. 5.**
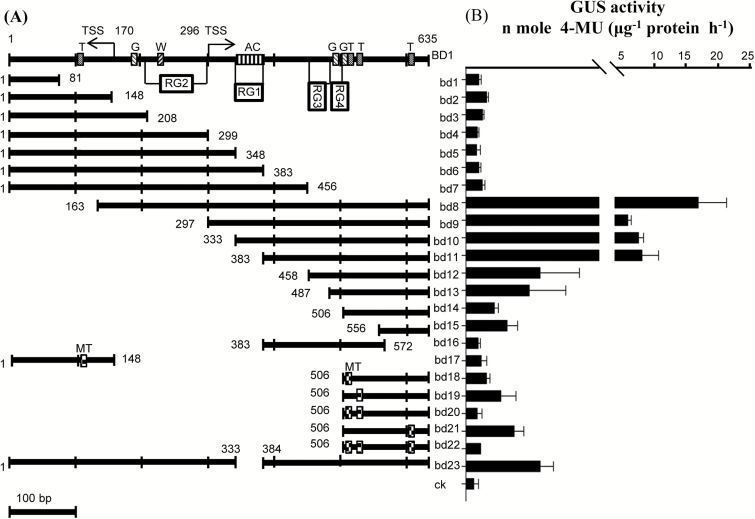
Truncation analysis of *cis*-elements of P_ZmBD1_. (A) Schematic representation of the *GFP*::mP_ZmBD1_::*GUS* constructs used for maize immature embryo transient transformation. TSS, transcription start site; T, TATA box; G, G box; W, W box; AC, AC-I/AC-II fused *cis*-elements; MT, mutated TATA box; RG, region; BD1, the intact bidirectional promoter P_ZmBD1_; bd1-bd23, the series of mutated bidirectional promoters of P_ZmBD1_; ck, maize immature embryos bombarded with empty vector. All mutated bidirectional promoters (mP_ZmBD1_) used *GUS* and *GFP* as dual reporter genes in one construct. (B) Quantification for GUS activity of mP_ZmBD1_ in immature embryos. Each bar represents data from between three representative independent bombardments, each bombardment contains nine immature maize embryos transformed with 1 μg plasmid of each constuct.

**Fig. 6.**
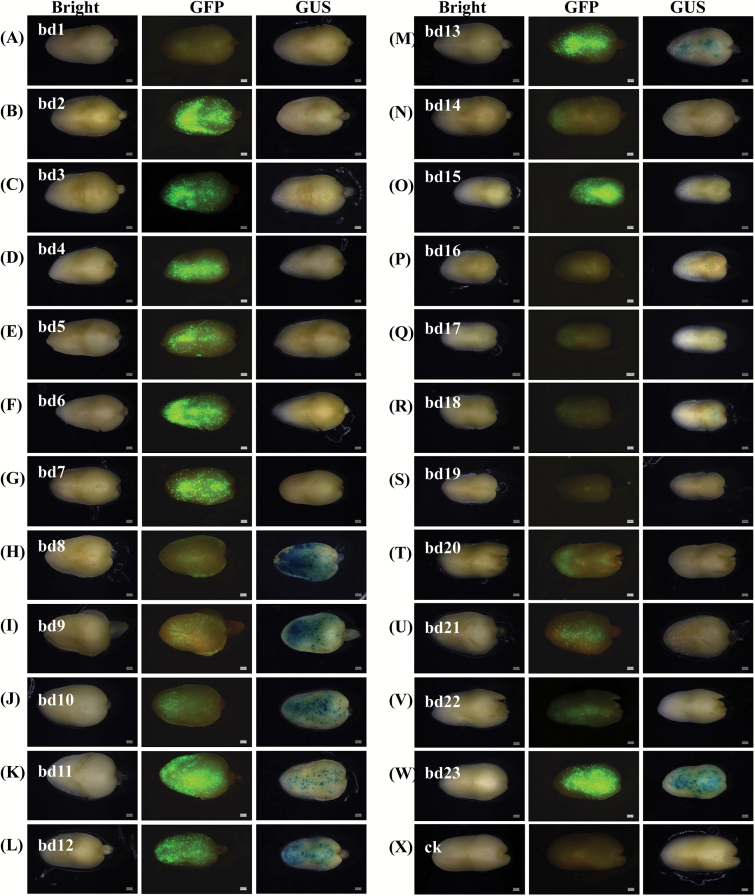
The activity of the different *GFP*::mP_ZmBD1_::*GUS* constructs assayed using maize immature embryo transient transformation. (A) to (W) represent the 23 constructs pbd1GG3 to pbd23GG3, respectively. Bright, pictures of the embryos were taken 24h after bombardment under a bright field using a Leica fluorescence stereoscope; GFP, pictures taken under dark field with ultraviolet light and a GFP filter following the bright field pictures; GUS, pictures taken after histochemical staining following the dark field pictures. Each construct was subject to two individual bombardments with six immature embryos; this figure shows one embryo. More information is shown in Supplementary Fig. S6. BD1, full length of the bidirectional promoter, and truncated from (A) 81 to 635, (B) 148 to 635, (C) 208 to 635, (D) 299 to 635, (E) 348 to 635, (F) 383 to 635, (G) 456 to 635, (H) 1 to 163, (I) 1 to 297, (J) 1 to 333, (K) 1 to 383, (L) 1 to 458, (M) 1 to 487, (N) 1 to 506, (O) 1 to 556, (P) 1 to 383 and 572 to 635, (Q) 148 to 635 and a point mutation in the TATA box, (R) 1 to 506 and a point mutation in TATA box 1, (S) 1 to 506 and a point mutation in TATA box 2, (T) 1 to 506 and point mutations in TATA boxes 1 and 2, (U) 1 to 506 and point mutations in TATA box 3, (V) 1 to 506 and point mutations in TATA boxes 1–x3, (W) 333 to 384, and (X) empty vector. Bars, 0.5mm.

To investigate the potential mechanism regulating a bidirectional promoter, a dual reporter gene transient system was used to test the transcriptional activity of a series of truncation, internal deletion, and point mutation constructs (Fig. 5A) of P_ZmBD1_. As shown in Figs 5 and 6, we found that some elements control the promoter activity and direction of transcription initiation. The two longer fragments, bd6 (positions 1 to 383) and bd7 (positions 1 to 456), produced a stronger GFP signal (Figs 5B, 6F, 6G) than the two shorter fragments, bd4 (positions 1 to 299) and bd5 (positions 1 to 348), (Fig. 5B, 6D, 6E). While the two even shorter fragments, bd2 (positions 1 to 148) and bd3 (positions 1 to 208), recovered the strong GFP-expression activity and showed GUS-expression activity (Figs 5B, 6B, 6C), but the 81bp fragment (bd1) lost the activity in both directions (Figs 5B, 6A). These results indicated that the common region (positions 348 to 383, RG1) shared by bd6 and bd7 functions as an enhancer in the GFP-expression direction, the common region (positions 208 to 299, RG2) shared by bd4 and bd5 served as a repressor regulating bidirectional expression, and RG1 has dominant epistasis to RG2. Sequence analysis showed that RG1 is an AC-I/AC-II fused element (Fig. 5A), which could interact with the G (positions 196 to 202) box to enhance gene expression (Hatton *et al.*, 1995). RG2 contained a W box (positions 229 to 235) (Fig. 5A). The W box was reported to interact with transcription factor (TF) OsWRKY71, which represses GA induction of the Amy32b promoter in aleurone cells (Zhang *et al.*, 2004). Furthermore, the region between bd2 and bd1 suggested that a TATA box (positions 100 to 103, TATA) plays a key role in regulating bidirectional expression (Figs 5B, 6A, 6B). This speculation was verified by the TATA box mutant (CACA) construct in which the bidirectional expression was dramatically decreased (Figs 5B, 6V).

Three truncated fragments, bd8 (positions 163 to 635), bd9 (positions 297 to 635), and bd10 (positions 333 to 635), displayed weak GFP-expression activity and strong GUS-expression activity (Figs 5B, 6H–J), the opposite expression activity to that of the shorter truncation fragments bd11 (positions 383 to 635) and bd12 (positions 458 to 635) (Figs 5B, 6K, 6L). bd13 maintained the promoter strength in the GFP-expression direction as in bd11 and bd12, while the activity in the GUS-expression direction decreased (Figs 5B, 6M). However, the bidirectional transcription activity decreased to a lower expression level when the truncated fragments were 129bp (bd14) long (Figs 5B, 6N). These results indicate that the common region (positions 348 to 383, RG1) shared by bd8, bd9, and bd10 functions as an enhancer in the GUS-expression direction, the common region shared by bd11, bd12, and bd13 (positions 458 to 487, RG3) functions as another enhancer in the GUS-expression direction, and the fourth region (positions 487 to 506, RG4) functions as another enhancer in the GFP-expression direction. Sequence analysis showed that the two fused elements ABRE/IRO2OS (positions 484 to 491 and 499 to 506) overlap two G boxes (positions 487 to 492 and 502 to 507) in RG3+RG4 that could interact with the AC-I/AC-II fused element in RG1 enhancement of transcription activity (Figs 5B, 6A). bd12, which contained two fused ABRE/IRO2OS elements, has strong bidirectional promoter activity (Figs 5B, 6L), while the activity decreased dramatically in one direction when one ABRE/IRO2OS (bd13) was broken (Figs 5B, 6M), and vice versa (bd14) (Figs 5B, 6N). Therefore, AC-I/AC-II and ABRE/IRO2OS are involved in regulating bidirectional activity.

Sequence analysis also identified three TATA boxes (box 1, 508 to 514; box 2, 529 to 534; box 3, 603 to 608) in bd14 (129bp) (Fig. 5A). The mutated box 1 led to the loss of GUS expression, but significantly increased the GFP signal (Figs 5B, 6O), while the mutated box 2 showed the opposite results (Figs 5B, 6P). The results with the mutated box 3 were similar to those of box 1 (Figs 5B, 6R). The fragments lost promoter activity when boxes 1 and 2 were mutated simultaneously or all three boxes were mutated (Figs 5B, 6Q, 6S). However, fragment bd15 (positions 556 to 635), which contains only box 3, had bidirectional promoter activity (Figs 5B, 6T) and fragment bd16 (positions 383 to 572), containing boxes 1 and 2, and lacking the bd15 region, just maintained GFP expression (Figs 5B, 6U). These results indicate that TATA box 1 is responsible for transcription initiation in one direction and antagonizes TATA box 2, which regulates transcription initiation in the other direction, and they are both are involved in coordinated bidirectional expression.

## Discussion

Maize is a critical food and feed crop that is also used as an efficient and safe plant bioreactor to produce industrial enzymes, antibodies, vaccines, and pharmaceutical compounds (Ramessar *et al.*, 2008). Moreover, metabolic engineering and trait stacking have been used in maize to modify complex metabolic pathways and to improve crop quality (Naqvi *et al.*, 2010). Embryo-specific promoters and efficient strategies are required to achieve these goals. In this study, we report the identification of a defensin-like protein bidirectional gene pair, *Def1* and *Def2*, which are expressed at high levels in maize seeds. The intergenic region of the gene pair functions as an embryo-specific bidirectional promoter, and the P_Zm*Def1*_ and P_Zm*Def2*_ function as embryo-specific promoters. Transient and stable transformation assays revealed that the polar promoters of the *Def1* and *Def2* genes function as strong and stringent embryo-specific promoters (Figs 3, 4, Supplementary Figs S1, S2). The 2.0-kb P_Zm*Def1*_ exhibited trace expression in leaves (Supplementary Figs S1, S2) and the 635-bp intergenic region of *Def1* and *Def2* functioned as a strong and stringent embryo-specific asymmetric bidirectional promoter sharing aluerone-specific characters, which we designated P_ZmBD1_ (Fig. 4, Supplementary Fig. S4). The P_ZmBD1_ bidirectional promoter could serve as promoter resources for maize and monocot biotechnology, which could be used for gene stacking and metabolic pathway engineering in maize and other monocot plant species.

**Fig. 7.**
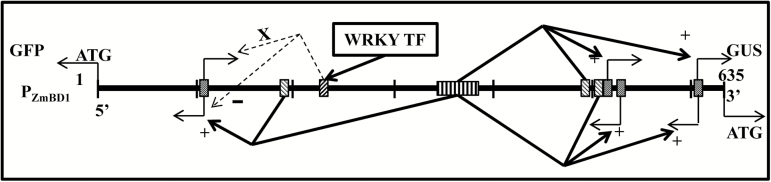
Model showing the potential mechanism regulating a maize bidirectional promoter. –, negative regulation of the core promoter activity; +, positive regulation of the core promoter activity; x, eliminates the activity of the bidirectional core promoter in one direction. WRKY TF, WRKY transcription factors. The core promoters contain a TATA box at the proximal end to ATG with low transcription activity in one or more directions forming the backbone of a bidirectional promoter. An enhancer-like AC-I/AC-II fused *cis*-element interacts with some *cis*-elements that positively enhance the bidirectional promoter activity, while WRKY TF binding to the W box down-regulates or eliminates the activity of one core promoter. Due to the number of core promoters and because the regulating effects at both ends are asymmetric, there has some differences in the promoter activity of P_ZmBD1_ in the two directions.

In vertebrates, studies of promoter sequences and TF binding sites showed that a small set of TFs interact with *cis*-regulatory sequences in bidirectional promoters and may constitute a simple mechanism for the regulation of bidirectional transcription (Li *et al.*, 2006; Lin *et al.*, 2007; Trinklein *et al.*, 2004). In plants, 1, 16, and 39 *cis*-regulatory sequences were shown to be overrepresented in bidirectional promoters of *Populus*, rice, and Arabidopsis, respectively (Dhadi *et al.*, 2009), but solid, direct evidence that would establish a clear model of bidirectional promoter regulation was not presented. Recent studies have revealed that bidirectionality is an inherent feature of promoters (Jacquier, 2009; Carninci, 2010; Wei *et al.*, 2011). Nascent transcripts in both directions at the transcription start site of a promoter were detected in almost equal amounts (Core *et al.*, 2008); however, most promoters produce final transcripts in just one direction. A potential model for bidirectional regulation of a promoter via the initiation, elongation, and termination steps of the transcription process may provide an explanation (Wei *et al.*, 2011). An assumed 5ʹ checkpoint in this model may control the transcription process between pause, elongation, and termination. With transcription from a promoter initiated equally in both directions, the RNA polymerase would pause at the 5ʹ checkpoint and epigenetic modification associated with the 5ʹ checkpoint would determine whether subsequent elongation or termination would occur. Transcript stability would then control the amount of transcript accumulation. Polar promoters display orientation preference, whereas bidirectional promoters produce stable transcripts in both directions; however, the mechanism that regulates bidirectional promoters remains unclear. Our study suggests that the mechanism of bidirectional transcription initiation of a bidirectional promoter involves one core promoter, located at both ends with relatively low bidirectional transcription initiation activity and *cis*-elements with various combinations of antagonistic or synergistic effects to negatively or positively regulate the level of transcription (Fig. 7). The two core promoters bd2 and bd15 in our model are additional evidence that bidirectionality is an inherent feature of promoters (Jacquier, 2009; Carninci, 2010; Wei *et al.*, 2011), and the W box interaction with WRKY TFs may serve as a 5ʹ checkpoint (Wei *et al.*, 2011). There may be other 5ʹ checkpoints near the other core promoter. However, the activity of all of the 5ʹ checkpoints can be eliminated by enhancer-liked activity so that a promoter develops a new characteristic: bidirectionality.

In this study, we demonstrated that two genes with similar functions were arranged in a head-to-head fashion with the 635-bp intergenic region serving as an embryo-specific, bidirectional promoter. The relative expression levels of two reporter genes driven simultaneously by the bidirectional promoter showed that the promoter strength was asymmetric. This gives rise to questions as to what the relationship between the two genes in the defense pathway is and whether the asymmetry is caused by differences in the responses of the two genes to signals during various stages of development, as part of a defense pathway, or in response to other factors. We also constructed a potential model in which core promoters with low bidirectional expression activity interacted with *cis*-elements, which conferred bidirectionality to the promoter, making it a so-called bidirectional promoter. A more detailed study of the role of the *cis*-elements is needed and is a subject of future research.

## Supplementary data

Supplementary data is available at *JXB* online.

Fig. S1. Comparative analysis of the tissue specificity of the polar and bidirectional promoters in transgenic maize plants.

Fig. S2. Comparative analysis of the tissue specificity of the polar and bidirectional promoters in transgenic maize plants.

Fig. S3. Comparative analysis of the expression patterns of the polar and bidirectional promoters during early developmental stages of transgenic maize plants.

Fig. S4. Analysis of bidirectional promoter tissue specificity in transgenic maize.

Fig. S5. Characterization of the tissue specificity and expression strength of the P_Zmdef2_ in transgenic maize plants.

Fig. S6. The activity of different *GFP*::mP_ZmBD1_::*GUS* constructs assayed using maize immature embryo transient transformation.

Table S1. Promoter sequences and cloning primers used in the study.

Supplementary Data

## Abbreviations:

Bd: bidirectional fragment
DAP: days after pollination
GFP: green fluorescent protein
GUS: β-glucuronidase
P: promoter
RG: region.
