# Supplementary material for: The intergenic region of the maize defensin-like protein genes Def1 and Def2 functions as an embryo-specific asymmetric bidirectional promoter
Source: J Exp Bot. 2016 Jun 8;67(14):4403–13. doi: 10.1093/jxb/erw226 (PMC5301941; doi:10.1093/jxb/erw226)
Supplement: Supplementary Data [file supp_67_14_4403__index.html]

The intergenic region of the maize defensin-like protein genes Def1 and Def2 functions as an embryo-specific asymmetric bidirectional promoter — The intergenic region of the maize defensin-like protein genes Def1 and Def2 functions as an embryo-specific asymmetric bidirectional promoter — Supplementary Data 

# The intergenic region of the maize defensin-like protein genes *Def1 and Def2* functions as an embryo-specific asymmetric bidirectional promoter

## Supplementary Data

Data files

- supplementary\_figures\_S1\_S6\_tables\_S1.pdf - Supplementary Data
