# Supplementary material for: The intergenic region of the maize defensin-like protein genes Def1 and Def2 functions as an embryo-specific asymmetric bidirectional promoter
Source: J Exp Bot. 2016 Jun 8;67(14):4403–13. doi: 10.1093/jxb/erw226 (PMC5301941; doi:10.1093/jxb/erw226)
Supplement: Supplementary Data [file supp_erw226_supplementary_figures_S1_S6_tables_S1.pdf]

The intergenic region of the maize defensin-like protein genes Def1 and Def2 functions as a embryo-specific asymmetric bidirectional promoter

Xiaoqing Liu, Wenzhu Yang, Ye Li, Suzhen Li, Xiaojin Zhou, Qianqian Zhao, Yunliu Fan, Min Lin, and
Rumei Chen

Supplementary Files

The following supplementary material is available at *JXB* online.

Fig. S1 Comparative analysis of the tissue specificity of the polar and bidirectional promoters in transgenic maize plants. Root, stem, and leaf tissues of trefoil-stage transgenic maize plants were used for GUS histochemical staining. Red arrow indicates trace GUS expression.

Fig. S2 Comparative analysis of the tissue specificity of the polar and bidirectional promoters in transgenic maize plants. Root, stem, leaf, and sheath tissues of large, trumpet-stage transgenic maize plants and tassels, silk, and pollen of transgenic maize plants were used for GUS histochemical staining. Red arrow indicates trace GUS expression.

Fig. S3 Comparative analysis of the expression patterns of the polar and bidirectional promoters during early developmental stages of transgenic maize plants. (A) Analysis of promoter tissue specificity using GUS histochemical staining in transgenic maize. (B) Analysis of the tissue specificity of *GFP*::P_{zmDef1}::*GUS* in transgenic maize. Red arrow indicates GUS staining sites in the aleurone layer and in embryos; Control tissues corresponding to the various developmental stages are shown on the right side of each photograph. DAP, days after pollination; bars = 1 mm.

Fig. S4 Analysis of bidirectional promoter tissue specificity in transgenic maize.

Fig. S5 Characterization of the tissue specificity and expression strength of the P_{Zmdef2} in transgenic maize plants. (A) Qualitative analysis of bidirectional expression activities of P_{Zmdef2} in transgenic maize seeds; Bright-I and GFP-I, intact seeds imaged in bright field mode and using a GFP filter set, respectively; Bright-L, GFP-L, and GUS-L, longitudinal sections imaged in bright field mode, using a GFP filter set, and in bright field mode after histochemical staining, respectively. Control tissues

corresponding to the various developmental stages are shown on the right side of each photograph; bars = 1 mm. (B) Quantification of GUS activities level during various developmental stages of seeds from transgenic maize plants. En, endosperm; Em, embryo.

Fig. S6 The activity of different *GFP*::mP$_{\text{ZmBD1}}$::*GUS* constructs assayed using maize immature embryo transient transformation. (A) to (W) represent the 23 constructs pbd1GG3 to pbd23GG3, respectively. (X), negetive control. Bars = 0.5 mm.

Table S1. Promoter sequences and cloning primers used in this work.

Figure S1

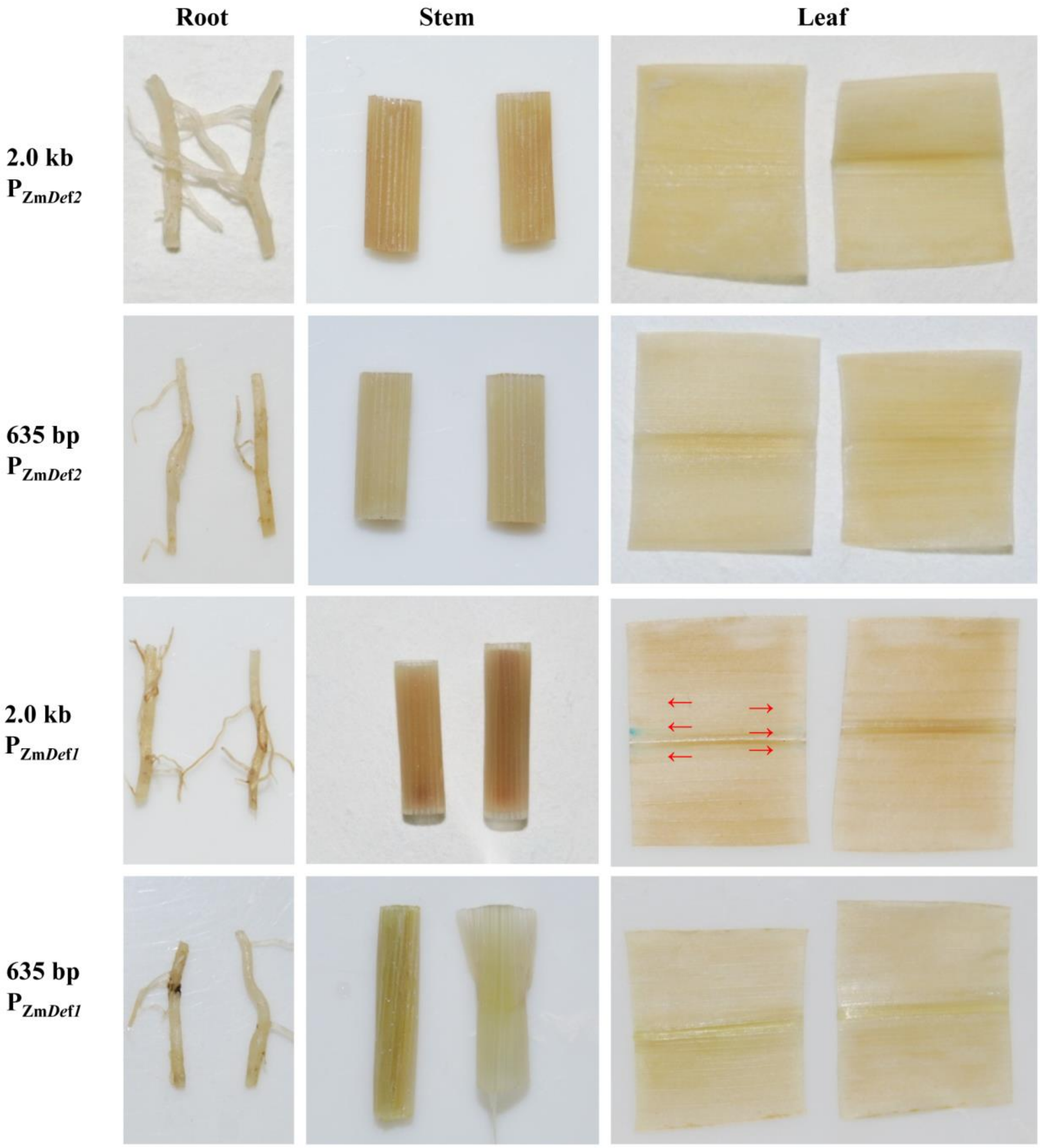

Figure S2

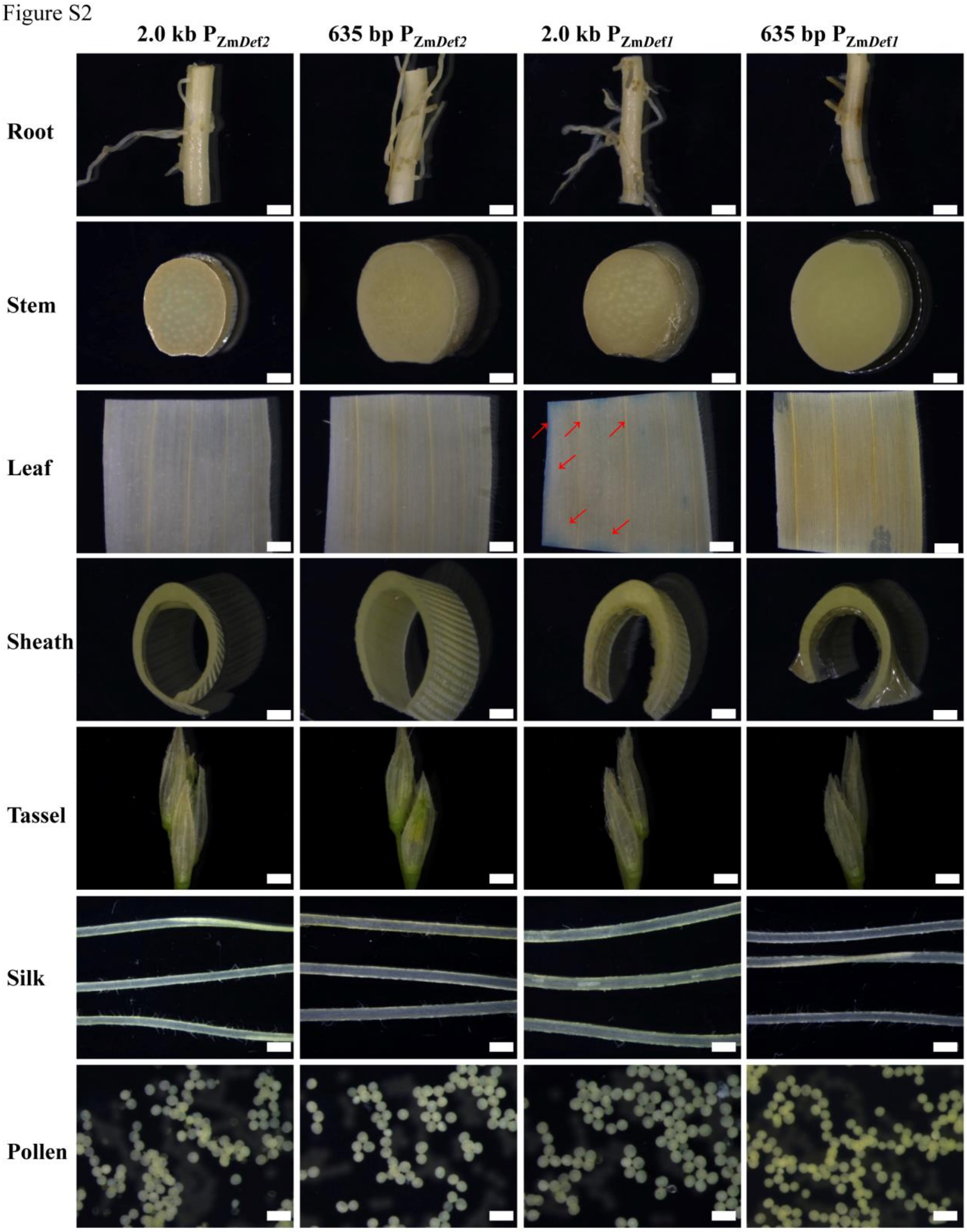
2.0 kb P_{ZmDef2}
635 bp P_{ZmDef2}
2.0 kb P_{ZmDef1}
635 bp P_{ZmDef1}
Root
Stem
Leaf
Sheath
Tassel
Silk
Pollen

Figure S3

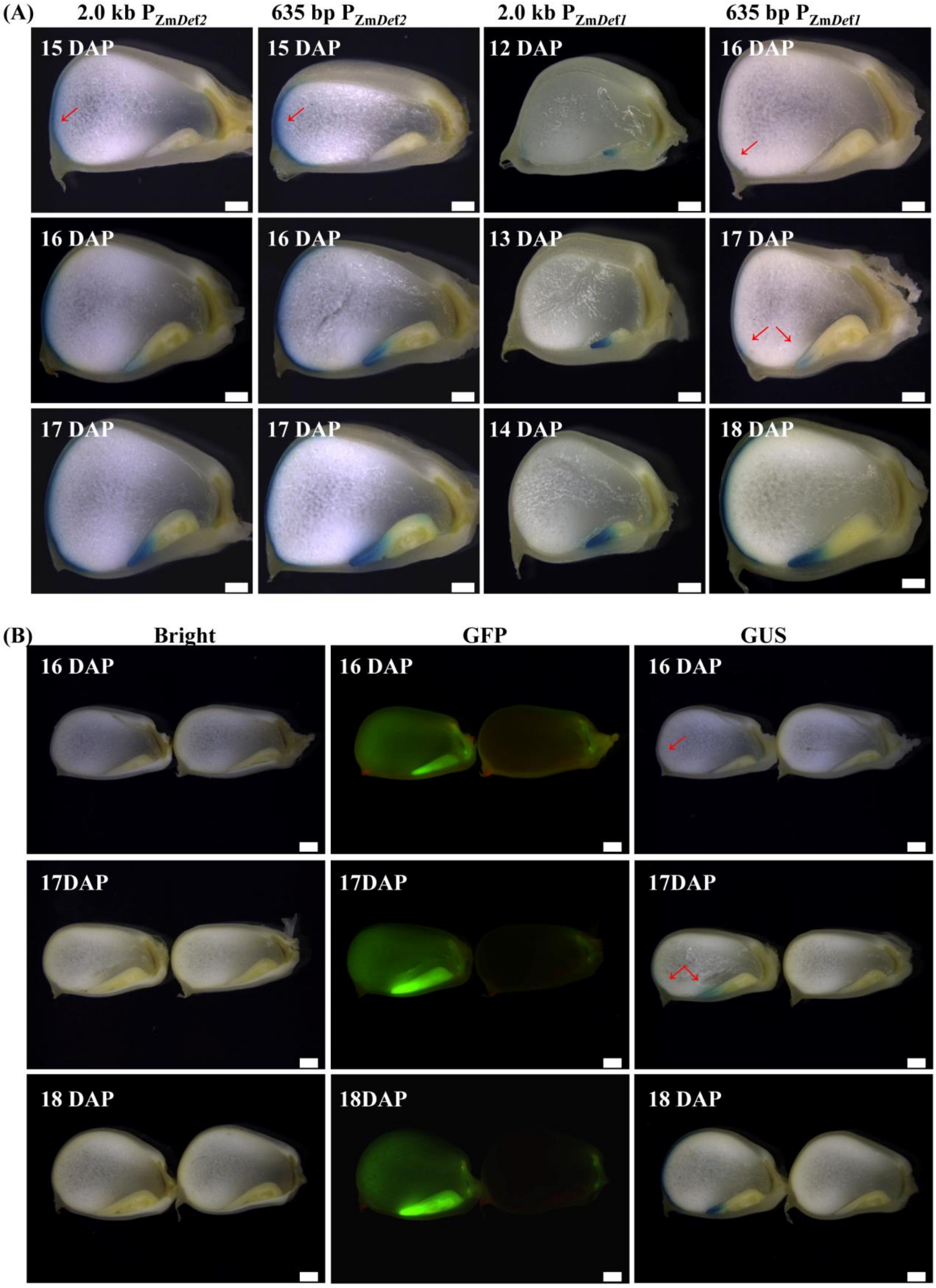
(A)
2.0 kb $P_{Zm\mathit{Def2}}$
635 bp $P_{Zm\mathit{Def2}}$
2.0 kb $P_{Zm\mathit{Def1}}$
635 bp $P_{Zm\mathit{Def1}}$
15 DAP
15 DAP
12 DAP
16 DAP
16 DAP
16 DAP
13 DAP
17 DAP
17 DAP
17 DAP
14 DAP
18 DAP
(B)
Bright
GFP
GUS
16 DAP
16 DAP
16 DAP
17DAP
17DAP
17DAP
18 DAP
18DAP
18 DAP

Figure S4

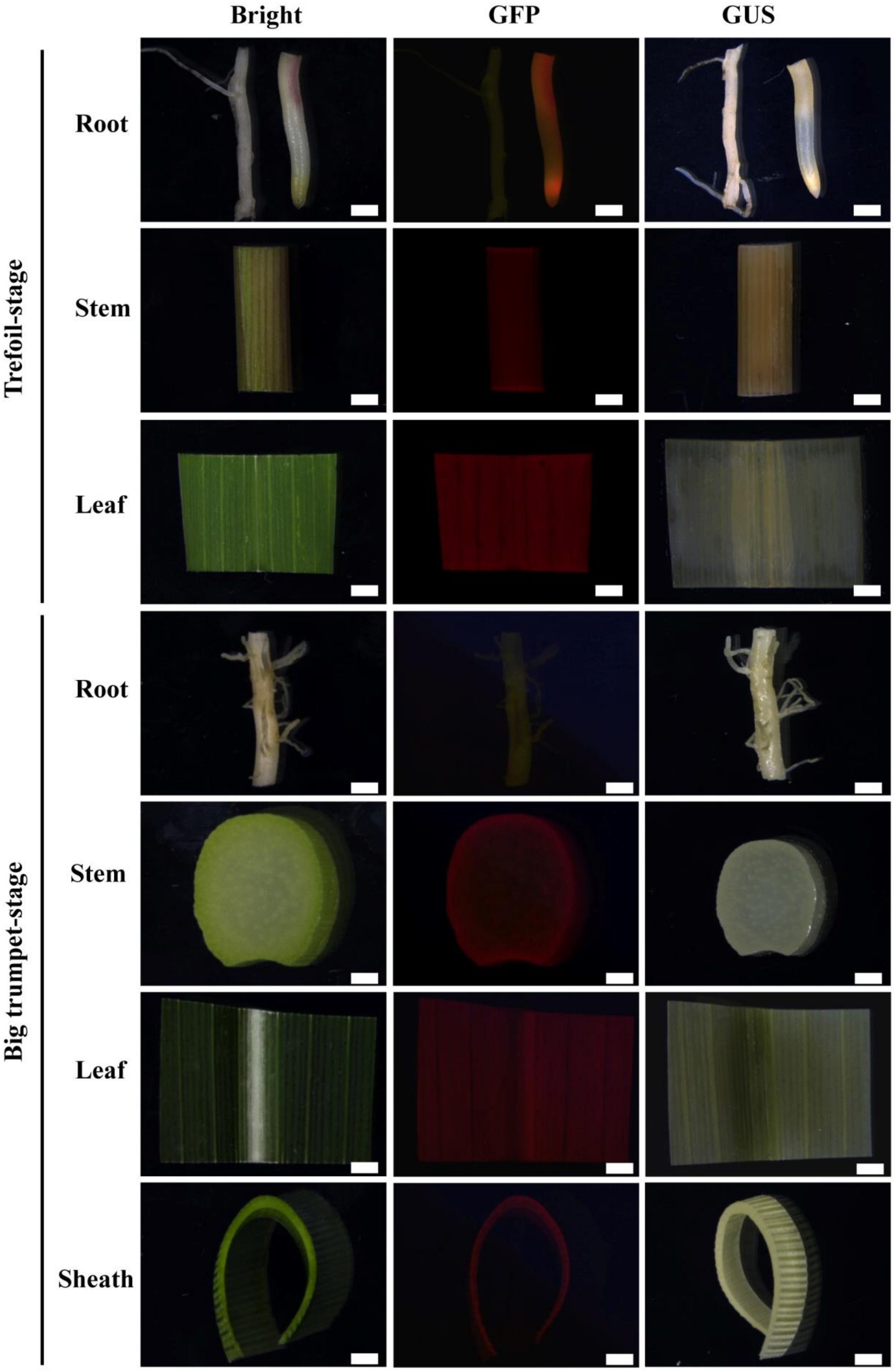
Bright
GFP
GUS
Trefoil-stage
Root
Stem
Leaf
Big trumpet-stage
Root
Stem
Leaf
Sheath

Figure S5

(A)

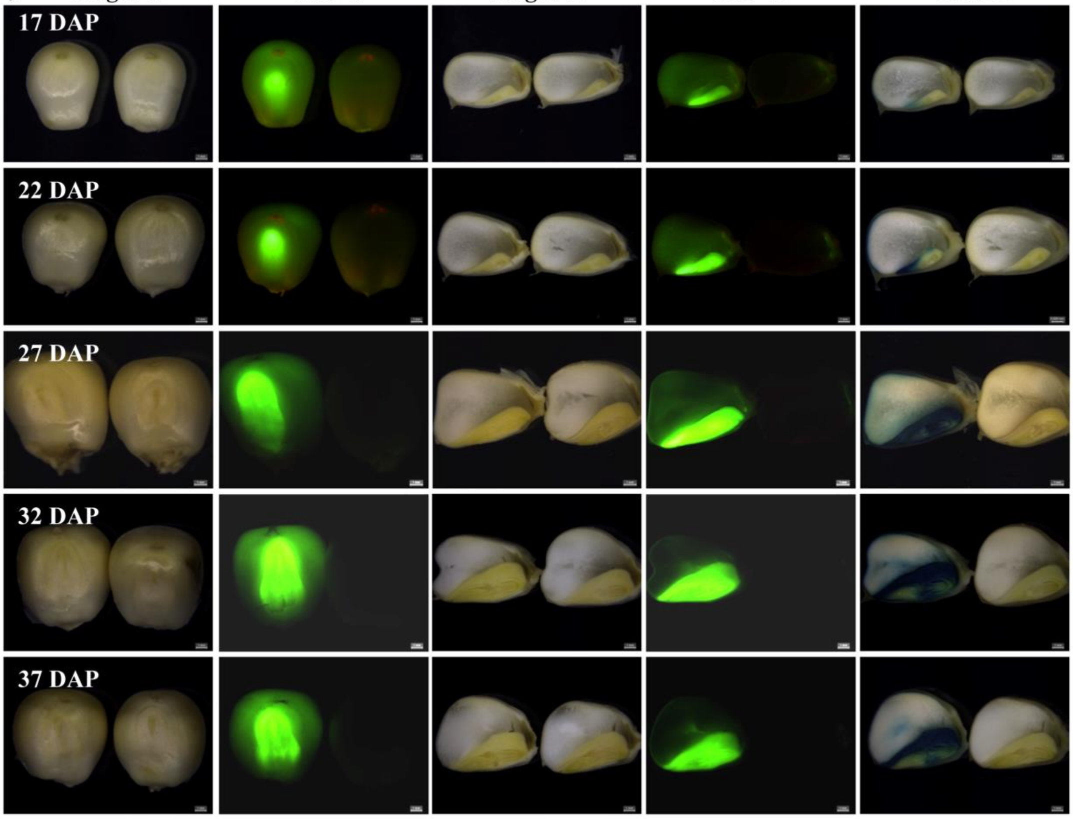
Bright-I
GFP-I
Bright-L
GFP-L
GUS-L
17 DAP
22 DAP
27 DAP
32 DAP
37 DAP

(B)

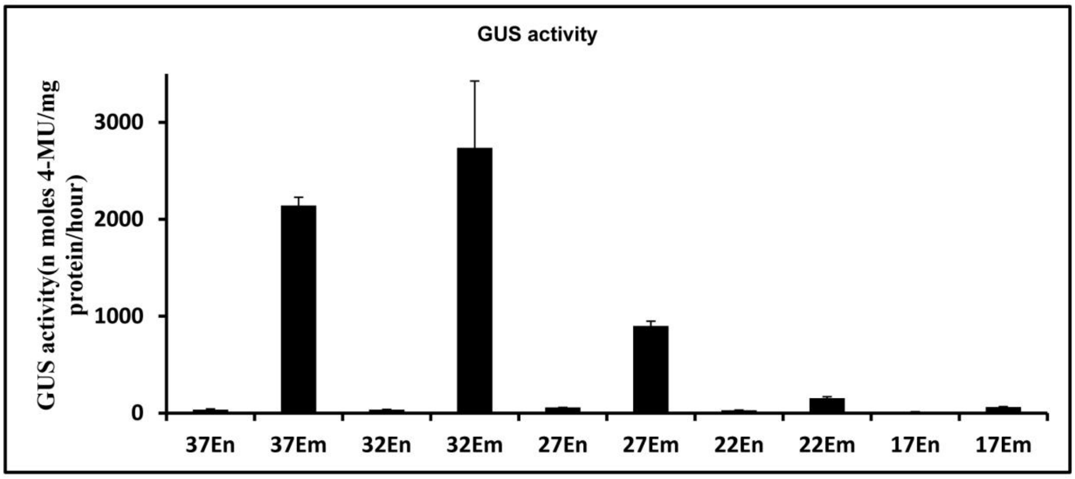
GUS activity
GUS activity(n moles 4-MU/mg protein/hour)
3000
2000
1000
0
37En
37Em
32En
32Em
27En
27Em
22En
22Em
17En
17Em

Figure S6

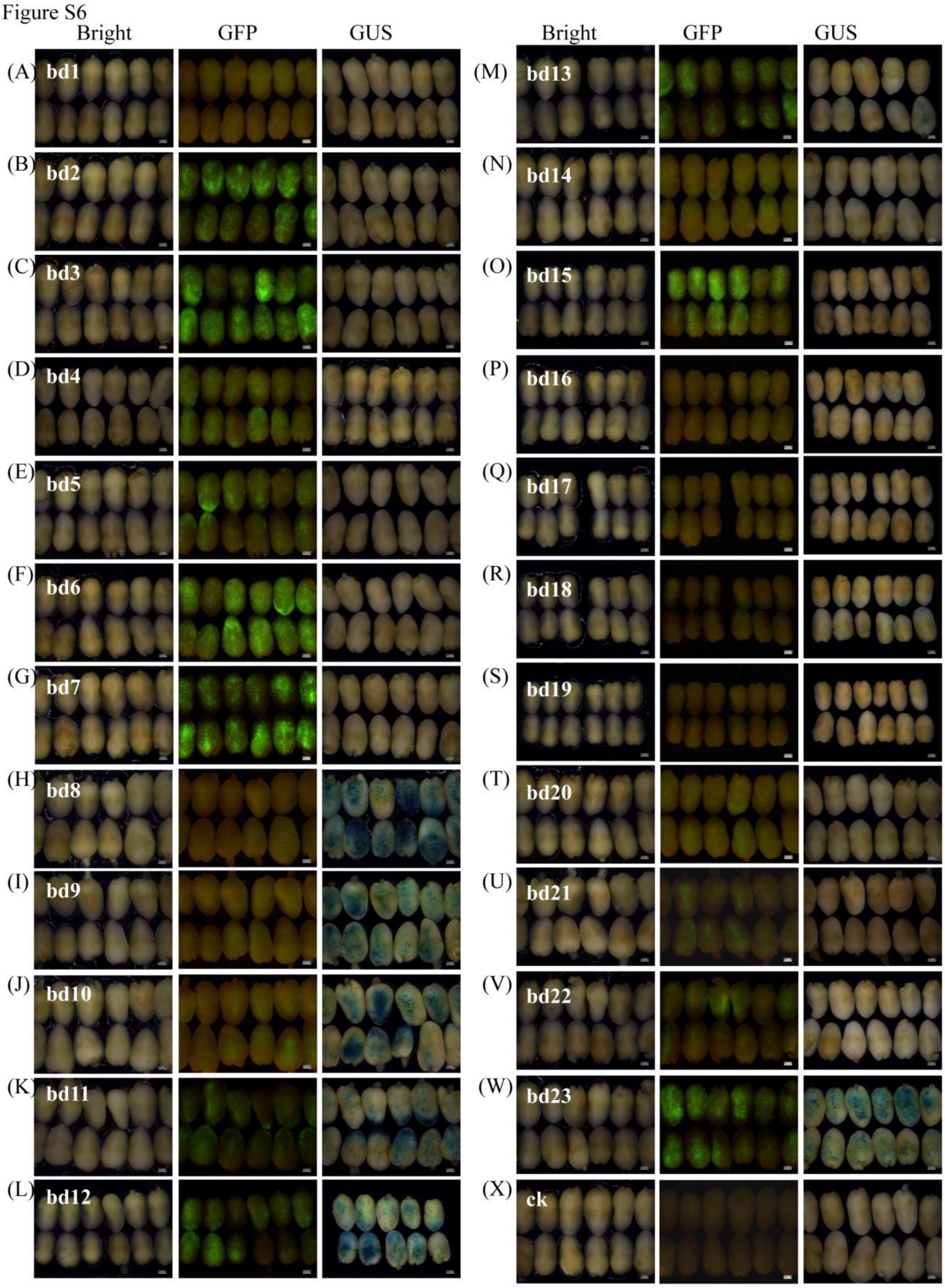
Bright
GFP
GUS
Bright
GFP
GUS
(A) bd1
(B) bd2
(C) bd3
(D) bd4
(E) bd5
(F) bd6
(G) bd7
(H) bd8
(I) bd9
(J) bd10
(K) bd11
(L) bd12
(M) bd13
(N) bd14
(O) bd15
(P) bd16
(Q) bd17
(R) bd18
(S) bd19
(T) bd20
(U) bd21
(V) bd22
(W) bd23
(X) ck

Table S1. Promoter sequences and cloning primers used in this work.

primers used for RT-PCR		
Def1	DEF1F	5-TGCTGCTCCTCATCGTCGTTGC-3
	DEF1R	5-TTGCCGCCGCCGTAGCCTTC-3
Def2	DEF2F	5-AGTCCAGGGCGACCGTGTG-3
	DEF2R	5-CGAGTGGTGCTGGCTCTTGC-3
Actin	AC200F	5-ATGTTTCCTGGGATTGCCGAT-3
	AC200R	5-CCAGTTTCGTCATACTCTCCCTTG-3
primers used for promoters clone		
2.0 kb $P_{Zm\mathit{Def1}}$	DEF1f	5-AAGCTTCGTCAGCAGTAGTTCTATTTCAG-3
	DEF1r	5-ATTCCGGCCGGCTCTGCTGCTTGTGC-3
2.0 kb $P_{Zm\mathit{Def2}}$	DEF2f	5-AAAAACTTTGGAGCCTAAACCTCTAAATTAAAG-3
	DEF2r	5-CTGCAGCGCCGGCCGGCGTGGATCGGAT-3
635 bp P_{ZmBD1}	DEF1f1	5-aagcttATTCCGGCCGGCTCTGCTGCTTG-3
	DEF2r	5-CTGCAGCGCCGGCCGGCGTGGATCGGAT-3

Promoter sequences:

>635 bp P_{ZmBD1}

attccggccggctctgctgcttgtgcttggtcgactactgatgtgggatgagcgtcagatcgtatgctggatgaccctgcctgcttgttggctcgcagat
ataggcgcggccgggcgcgcggccggcggtggaccgtggacggcgcgcgcatgcaagagcggcgacgcgtggcgagctgcggcggccgcg
catgcgcacgtggctgcccgcacgggctgcatgcgcgccaggtcagctaggggcagcacgctgagccgagtgcgtgccgtacgtagcgtcgcca
gcagctgattctaactttctgttctaaactttctaggtgcatggtaccgatccacccacccacccctcctcgcctgtctgccacgcacgggatcggagga
ggttgtccttgacgtcgtctcgagtcgacgacgagctcgcagcaccggccgctagctggcctcacgcggtgcggtggcagcaggtggcagggag
cgagccgccacgtgccgcgctgccacgtgagtataagtaccctgctctgctatatagagctcgcaactcgctggccatagatccattgattgatcgga
cggacgcgcgtctccggtcccgtccgtaataacactgatccgatccacgccggccggcg

>2.0 kb $P_{Zm\mathit{Def1}}$

ttcgtcagcagtagttctatttcagagaatgaaaggagctctgtcagatgcccctaactttctcgtgaactgtacgtggaagcacgcccgtagttgctgg
ctgtgtgaatctgattctggatgcaacgaagaatagtaaaaatgcagcgtctgttatttattagcgtgttgctgaactggactggaatgatgcagcttgga
acgacgcggtggaggaggcgtgccagcgcggcgtggcggtgatcgcagagcccggcggcagcatccgggaccaggacgccgtggagtgctg
caacaagtacggggtggccctcgtcttcaccggcgtccggcacttcaggcactgatcgacggcgacggtggcagcggagaccgtgacgacggac
gatgggagggacctctatatagctgcgtcgatctggccggtggctggaaggaaggatctggcgtttcatcccagcgctgatgccggctggcgtttctt
ggctactagggagactctgatggtgctggatactcagtggcaaagagtagagaaagatacaaatggcagaactgttcactgtattgcaggaactatag
tttatatacaagaatataagtgatttttaataatgtgtccgttggaaataactgttagtagttgatcacatgagataacatggtatacaaccaaatgtcgcctc
tcgagaggatgggtcggtgggtcccggtttgtatatatgacgtcgtcagcctggcaagaggttgattgagcttaatactaactcttgcggtggatggaa
atcggaaacggtcgctgaccggtgggatcgcacagtctgctgctagaagcatgcaaaggctacatgccgtgttattattgttattaatatttattgctgtc
acgttacatgacaccacattacatgcatgcacggtacgtaggttgtgcacagctagccaagggtgttacttagtactaccacagttcagttctggcaagc
caagggtgtttcaagagctttgcttagcacgccttctggcacctgcagtagcggaggtggcagtagccggcggtccacccgccgtcctccttgacgc
actcgttgctgcagaggcggtcggagatgcaggggaacgagtggtgctggctcttgcccatgcacacccgggcctccgccaccatcgtcgtcgtcg
tcccgccgtcgtagtctgtgcgtggatagcggaaggaggaagagtccatgaggcagcagaaattaagaataatacgtgaattaaatcaacgaataaa

tcagtatgcttttttttattacgtgagagcaggagcagcgccaggaggagcgcgcacacggtcgccctggacttgatgagctccatcgccggccggc
gtggatcggatcagtgttattacggacgggaccggagacgcgcgtccgtccgatcaatcaatggatctatggccagcgagttgcgagctctatatag
cagagcagggtacttatactcacgtggcagcgcggcacgtggcggctcgctccctgccacctgctgccaccgcaccgcgtgaggccagctagcg
gccggtgctgcgagctcgtcgtcgactcgagacgacgtcaaggacaacctcctccgatcccgtgcgtggcagacaggcgaggaggggtgggtgg
gtggatcggtaccatgcacctagaaagtttagaacagaaagttagaatcagctgctggcgacgctacgtacggcacgcactcggctcagcgtgctgc
ccctagctgacctggcgcgcatgcagcccgtgcgggcagccacgtgcgcatgcgcggccgccgcagctcgccacgcgtcgccgctcttgcatgc
gcgcgccgtccacggtccaccgccggccgcgcgcccggccgcgcctatatctgcgagccaacaagcaggcagggtcatccagcatacgatctga
cgctcatcccacatcagtagtcgaccaagcacaagcagcagagccggccggaat

>2.0 kb $P_{Zm\textit{Def2}}$

aaaaactttggagcctaaacctctaaattaaagtttgattcagatgattgtcgaactaatcagcaaatcgtgacctgacatcatgaatagtttatgctacaat

ccagtttaaataattatacaccttcaaactgtacacataaaatgaatatgacaagttgtacacactacttgatatgtctctcaagatgtctacgtatacacca

atatgccttcaagctgtagacatacagcagcaccagtgtaataacccccgatgagcacgtacgtgacaagaaaaatcgagagaacgccgacacgag

aaagcgagaacatgcaccgtccacaccagtgtaactcccgatgagcatgtatgtgacaagaaaaatcgagagaacgcgacatggctcccatcgatc

gtagcggccggcgggccgtgttggcacgttgctgacttacattaggataaattagacattaggattacatagcacgtatctttatcgatctcgaaacaatt

tcacatatttaacagccatggaaaaaatataaaaattgtattacacattattacgtacaggcaaatacttggtgtgagctagcacaagctgggataaacca

agcaaaaggtcgttaatagtgtaacttggatgcaaacttgtcatagttattgatataaaataaactgttgtcgctcttaaatttttcgataagagtatacgaac

atcaggacatgtatataatactgcatgcacgtgtttattgtcaagatgtcatgaccatcatcaaagcgtggcggccggatgcatgggtggacgtcgacg

acacagctcatgagtactcgaacgacagacgacgacgtataggaacgatggccgtgcatgctagctgcggcgtccacacagctcggcgcgcgtac

gcgtacatgtggtgcattattgcatgtagtgtagacgtatagtagtacatatttagcgaccggccacgacgtcgatccttattaatttaattaaaggccgat

atagctagcttgctagatcctagagcctagctagctagcactccctgatgcacttgcactggcgcatgatgccgtcgcagttgccgccgccgtagccct

cctgcaagcacacctgcgcgcagttgtggtcggacatgcagaccccccttgaagcccgcgctccggcgccggcacacgcgcgcctccgccaacgg

cgccgcctcctctgcatgcattgcaggcacgcgtagtacgcatgatgatgtgtgcgtgtgtatatatatgttagtggtgtagctatagtgatagtgacgta

cgcgcaacgacgatgaggagcagcaggaggacggccacctgcggcgtcgccaccttcctgatcgtccacatattccggccggctctgctgcttgtg

cttggtcgactactgatgtgggatgagcgtcagatcgtatgctggatgaccctgcctgcttgttggctcgcagatataggcgcggccgggcgcgcgg

ccggcggtggaccgtggacggcgcgcgcatgcaagagcggcgacgcgtggcgagctgcggcggccgcgcatgcgcacgtggctgcccgcac

gggctgcatgcgcgccaggtcagctaggggcagcacgctgagccgagtgcgtgccgtacgtagcgtcgccagcagctgattctaactttctgttcta

aactttctaggtgcatggtaccgatccacccacccacccctcctcgcctgtctgccacgcacgggatcggaggaggttgtccttgacgtcgtctcgagt

cgacgacgagctcgcagcaccggccgctagctggcctcacgcggtgcggtggcagcaggtggcagggagcgagccgccacgtgccgcgctgc

cacgtgagtataagtaccctgctctgctatatagagctcgcaactcgctggccatagatccattgattgatcggacggacgcgcgtctccggtcccgtc

cgtaataacactgatccgatccacgccggccggcg
